# Professional Recreation

**Published:** 1902-08

**Authors:** 


### Editorial.

### PROFESSIONAL RECREATION.

THE mid-summer season brings to all workers a desire to throw off the trammels and grind of daily labor and seek somewhere on mountain, by lake or ocean, a change for a few days or weeks from the incessant treadmill, the lot of the majority. The strenuous life of the modern world stands in constant antagonism to mental and physical recuperation. Even the restful hours of sleep must be invaded in order that ambition may be satisfied. Night is turned into day and day into night, that the activities of the world may be in constant motion.

The question is oftentimes suggested, What is to be the end of all this? Is it possible for the human mind to continue this incessant strain? Will there not come a period when the mental faculties, overburdened, will degenerate in individuals and nations, and civilization suffer a relapse and reversion to original conditions? The lessons of history are full of meaning in this direction, and those who can grasp the profound significance of this may readily prophesy that the present era, marked as it is in progress, may be simply the precursor of another in which mental decadence may be the lot of the, at present, advanced civilizations.

These thoughts, while somewhat pessimistic, are worthy of greater elaboration than it is possible to give here, for they include the often-discussed topic, the rise and fall of nations, but they should receive consideration by those in our profession who labor from early morning to late at night, day in and day out, and imagine they are doing the best possible for themselves and their families. It is a fatal mistake, and one that many dentists are constantly making.

There are but few occupations more injurious to health, when care is not taken, than dentistry,—certainly not among the so-called professions. The confinement in unhealthy environments, the continual mental and physical strain, the exacting character of the hourly and daily operations, will sooner or later break down the most vigorous constitution if continued without systematized interruptions. The penalty of continued violations of natural law must be paid to the uttermost farthing.

Will the short respite taken during mid-summer prove sufficient to maintain a healthy equilibrium? This will greatly depend upon how this is improved, and it is difficult to propose any method of recreation that will suit all conditions. We are all differently organized, mentally and physically, but it may be assumed that to the dentist any change that takes him out of his daily surroundings will be beneficial.

In the opinion of the writer it is a mistake to transfer the busy man of affairs to some secluded spot where nothing exists to attract attention but the beautiful mountain scenery, and nothing to fill up his hours but a game of whist or billiards. The experience becomes an unendurable monotony, with a break-down or an incessant feeling of acute depression as a result of this misdirected effort. Neither the mind nor the body, while constantly in training, can suddenly cease the hourly activity and lapse into perfect indolence, without danger.

The dentist belongs to the before-mentioned class. When first out of his cage, he feels the joyous freedom from responsibilities, but this is not lasting. He has been trained to a system, and he cannot longer be happy without it. At first the feeling is to get away from people. He resorts, by inclination, to the mountains or secluded sections. He thinks he needs most to commune with nature without any of the frills of civilization. Is this the best for this class? The life of the average dentist is too much secluded, not so much from people, for of these he sees too many, but from that broader world of which he must know to avoid falling into the narrow ruts of his own constantly ploughed field. He must get out upon the level sward, that he may become more appreciative of the world with which he is surrounded.

The thoughts heretofore given must have frequently entered the minds of the dental fraternity in all our large cities, for there more than elsewhere has the fact that the dentist needs a change grown from year to year, until few offices are to be found open during August, and many close through the two mid-summer months. In the smaller towns and villages it is surmised that the work goes on continuously throughout the year.

The unwisdom of this becomes apparent to the individual when too late to be remedied, and long before threescore years and ten have been reached he has become prematurely aged and frequently a confirmed invalid.

Dentistry properly followed is not an unhealthy occupation. The sanitary conditions of the office must be carefully guarded, and then the individual should, with equal care, guard himself. He should never allow the temptation to overwork to dominate him. Many in the memory of the writer have filled early graves because of the insane desire to cut the hours of sleep and make the day of work from fifteen to twenty hours long.

The remedy for this seems to be to have fixed hours for labor and hours for recreation. While the summer holiday is excellent, the daily periods of cessation from labor are better. An active walk in the sun for an hour in the morning, before commencing the serious tasks of the day, is very invigorating, and a half-hour in the sun at mid-day will stir the circulation and prove an excellent antiseptic antagonist against the multitude of micro-organic elements contracted during the morning's work. Then close all labor at a fixed hour in the afternoon. The country practitioner will naturally say, If I follow this advice, what will become of my mechanical cases? These must be prepared in the evening. This is unquestionably one of the serious features of the dental practice in small places. It has been largely overcome by city practitioners, but no remedy has been found for those isolated from the active centres. The practice there ordinarily will not warrant the expense of an assistant, and he of the smaller place must do the work himself.

The time will come, indeed is already here, when dentistry will be divided into specialties. These will be classed as operative dentist; the general dental mechanician, who will take all cases of prosthetic work and assume the responsibility of them in the mouth; the "bridge-worker," who will confine his labor to that important branch. The oral surgeon will do nothing but care for the important surgical operations and the administration of anæsthetics, and eventually, doubtless, oral prophylaxis will be cared for by men and women, confining their labor exclusively to removing accretions upon the teeth. These all have now a place with the exception of the last, and when fully established the hours of labor need not be prolonged to the detriment of health, as at present.

The present month of August will find a large number taking advantage of the period to gain relaxation by mingling in the various conventions in this country and Europe, some of which will have ended their labors before this reaches our readers. This is beneficial, but is not all that is desirable. To what extent these conventions may benefit dentistry is, at this writing, problematical. In all probability the effect, if any, will not be immediate. Great changes are not to be expected or desired. It is the general uplift that has been given it and which will continue to be given by these annual gatherings that makes their coming together of vital importance.

These mid-summer months are not productive of the "silly season," as some choose to regard them. They may be all of this to many, but to those who are wise they may be made a period of recuperation, mentally and physically, and thus add new life to the individual and additional vitality to the dental profession, for without an improvement in the minor parts advancement is hopeless for the entire body.

